# Synthesis and Physical Characteristics of Undoped and Potassium-Doped Cubic Tungsten Trioxide Nanowires through Thermal Evaporation

**DOI:** 10.3390/nano13071197

**Published:** 2023-03-27

**Authors:** Po-Heng Sung, Hsi-Kai Yen, Shu-Meng Yang, Kuo-Chang Lu

**Affiliations:** 1 Department of Materials Science and Engineering, National Cheng Kung University, Tainan 701, Taiwan; ss87316@yahoo.com.tw (P.-H.S.); n56091302@gs.ncku.edu.tw (H.-K.Y.); n56074287@gs.ncku.edu.tw (S.-M.Y.); 2 Core Facility Center, National Cheng Kung University, Tainan 701, Taiwan

**Keywords:** cubic tungsten oxide, thermal evaporation, nanowires, photoluminescence, doping, field-emission

## Abstract

We report an efficient method to synthesize undoped and K-doped rare cubic tungsten trioxide nanowires through the thermal evaporation of WO_3_ powder without a catalyst. The WO_3_ nanowires are reproducible and stable with a low-cost growth process. The thermal evaporation processing was conducted in a three-zone horizontal tube furnace over a temperature range of 550–850 °C, where multiple substrates were placed at different temperature zones. The processing parameters, including pressure, temperature, type of gas, and flow rate, were varied and studied in terms of their influence on the morphology, aspect ratio and density of the nanowires. The morphologies of the products were observed with scanning electron microscopy. High resolution transmission electron microscopy, X-ray photoelectron spectroscopy, and X-ray diffraction studies were conducted to further identify the chemical composition, crystal structure and growth direction of the nanostructures. Additionally, the growth mechanism has been proposed. Furthermore, we investigated the potassium doping effect on the physical properties of the nanostructures. Photoluminescence measurements show that there were shorter emission bands at 360 nm and 410 nm. Field emission measurements show that the doping effect significantly reduced the turn-on electric field and increased the enhancement factor. Furthermore, as compared with related previous research, the K-doped WO_3_ nanowires synthesized in this study exhibited excellent field emission properties, including a superior field enhancement factor and turn-on electric field. The study reveals the potential of WO_3_ nanowires in promising applications for sensors, field emitters and light-emitting diodes.

## 1. Introduction

To keep Moore’s law alive for the next generation, electronic devices have become lighter, thinner and faster. However, conventional lithography has gradually reached its limitation; it has become much more difficult to fabricate nano-scale circuits through a top-down approach. On the other hand, bottom-up approaches, such as nanostructures synthesized by physical or chemical methods, have brought much more potential and possibility.

Furthermore, nanomaterials with dimensions under 100 nm have several unique properties that bulk materials do not possess. For example, the reflectivity can be less than 1% as compared with bulk materials [1] and can be implemented in optoelectronic or optothermal devices. Nanomaterials may have different structures where the quantum confinement effect appears and alters some properties, such as the band gap [2]. Certain new nanomaterial properties may provide the key to solving current technological challenges.

Transition metal oxide nanomaterials, including zinc oxide (ZnO) [3,4], titanium oxide (TiO_2_) [5], copper oxide (CuO) [6,7], and vanadium oxide (VO_2_) [8], have drawn attention for a variety of applications owing to their diversity of morphology, stability, catalytic activity and conductivity. Among them, tungsten oxide (WO_3_) is one of the most popular materials in optoelectronic devices [9,10], photodetectors [11], and gas-chromic fields [12,13]. Since WO_3_ has a shorter bleach color transition time, obvious color contrast, and stability, it has been developed to be applied in smart widows, sensors [14], and monitors.

Compared with bulk materials or thin films, 1-D nanostructures such as nanowires (NWs) have many excellent properties, including a high surface to volume ratio, dimensions comparable to the Debye length, and high crystallinity [15,16,17]. A number of methods have been utilized to synthesize tungsten oxide 1-D nanostructures, such as thermal oxidation, thermal evaporation, chemical vapor deposition, hydrothermal reaction, electrochemical techniques, using intercalated polyaniline, and a solution-based colloidal approach [10,18,19,20,21]. Of them, thermal evaporation could be the most attractive technique, with the advantage of fabricating various tungsten oxide nanostructures at lower temperatures than other methods. Additionally, thermal evaporation is currently the most stable and fast process in the synthesis of tungsten oxide nanostructures. A horizontal tube furnace is utilized to anneal the precursor powder; subsequently, precursor vapor is sent to the substrate by gas transport for deposition.

In this work, we synthesized very uniform and unique cubic tungsten trioxide nanowires through thermal evaporation by controlling certain parameters, including the introduced gas, pressure, and substrate position. We investigated the effect of the field emission property on the morphology and proposed the growth mechanism of the NWs. Since the chromism properties of tungsten oxide have been widely studied but its luminescence and field-emission properties have rarely been explored, our measurements are focused on the last two properties. Potassium is an interesting dopant for tungsten nanomaterials and has been extensively studied [22]. Numerous properties of tungsten nanomaterials are enhanced after the doping process. Additionally, we discussed the potassium doping effect on both the nanowire morphology and properties.

## 2. Materials and Methods

**Figure 1:**
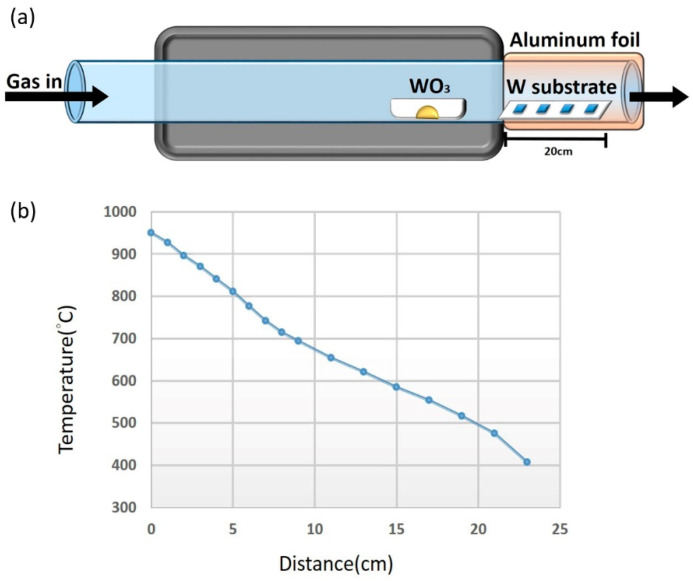
Experimental process. (**a**) Schematic illustration of thermal evaporation technique to synthesize WO_3_ NWs. (**b**) Temperature versus substrate position diagram measured with a thermal couple.

Synthesis of tungsten oxide nanowires on tungsten (W) substrates was conducted in a horizontal tube furnace through a thermal evaporation technique without catalyst. An alumina boat containing 0.5 g tungsten oxide (WO_3_) powder (99.9%, Alfa Aesar, Ward Hill, MA, USA) was placed at the center of the furnace, as shown in Figure 1a. The W substrates were cleaned in acetone, isopropyl alcohol, and deionized water sequentially by sonication in order to remove organic and inorganic substances on the substrates. Then, the substrates were placed at different temperature zones away from the heating zone by 1–20 cm. Figure 1b shows the temperatures of these zones, which were measured by a thermal couple. To maintain the temperature, the end of the quartz tube was covered with multiple layers of aluminum foil. The temperature of the furnace was raised to 1050 °C at a heating rate of 30 °C/min and the pressure was pumped down to 0.1–1 torr by a rotary pump. Argon (Ar) (99.9%, Yun-Hai Company, Tainan, Taiwan) at 5–50 sccm was introduced into the quartz tube depending on the desired pressure and doping concentration. After the duration of 1.5–6 h, the furnace was cooled down to room temperature.

In the potassium-doped (K-doped) WO_3_ NWs part, potassium iodide (KI) powder (99.5%, Sigma-Aldrich, St. Louis, MO, USA) was used as a source of doped K and was placed at the front of the furnace. The pre-made substrate grown at 700 and 750 °C with WO_3_ NWs were placed downstream of the furnace at 500 °C. Ambient pressure was kept at 0.1 torr, the carrier gas Ar was 10 sccm and the reaction time was 20 min, prior to cooling down to room temperature.

To understand the factors that influence the growth and doping of tungsten oxide nanowires, processing parameters including the temperature, pressure, and introduced gas were varied, and their effects were studied. The morphology and structure of the NWs were observed with a field emission scanning electron microscope (FE-SEM) (Hitachi SU8000, Tokyo, Japan). The composition, atom arrangements, and crystallinity were characterized through X-ray diffraction (XRD) (Bruker AXS Gmbh, Karlsruhe, Germany) and high-resolution transmission electron microscope (HR-TEM) (JEOL ARM200F, Tokyo, Japan) equipped with an energy-dispersive X-ray spectrometer (EDX). Also, the composition analysis of the materials was conducted with X-ray photoelectron spectroscopy (XPS) (ULVAC-PHI 5000 Versaprobe, Chigasaki, Kanagawa, Japan).

Field emission and photoluminescence properties were studied. The field emission measurements were conducted with a source measure unit (SMU) (Keithley 237, Cleveland, OH, USA) applied at 1100 V in a vacuum chamber. The working distance between the cathode and anode was 60 and 100 um; the receiving area of electric current was 0.00785 cm^2^. Then, we analyzed the J-E patterns and F-N distributions to evaluate the field emission characterizations. The photoluminescence characterizations of WO_3_ NWs were obtained by a Micro-PL (Jobin Yvon/Labram HR, Paris, France) with the main incident source wavelength of 325 nm at the temperature of 300 K.

## 3. Results

### 3.1. Synthesis and Growth Mechanism

**Figure 2:**
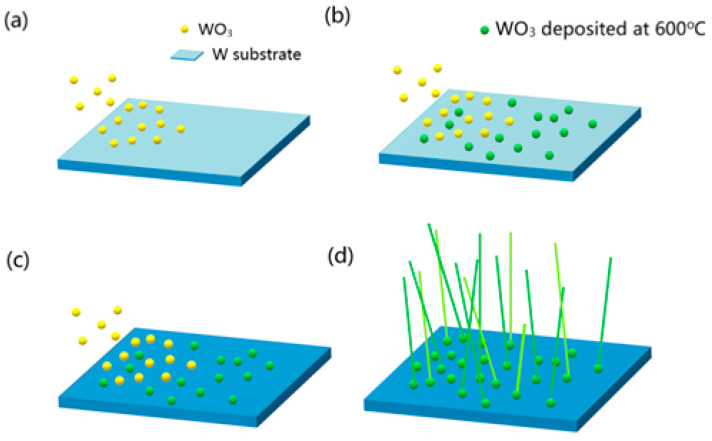
The growth mechanism of thermal evaporation. (**a**) The tungsten trioxide vapor moved to the substrate by the carrier gas. (**b**) The tungsten trioxide vapor effectively collided with the tungsten substrate and nucleated on the substrate. (**c**) Formation of a dark blue film of tungsten oxide on the substrate. (**d**) Tungsten trioxide nanowires were grown from the protrusions of the film as nucleation points.

The mechanism of the growth by thermal evaporation is shown in Figure 2. Firstly, the tungsten trioxide vapor moved to the substrate by the carrier gas in Figure 2a. After the tungsten trioxide vapor effectively collided with the tungsten substrate, it was adsorbed on the substrate and resulted in a heterogeneous nucleation, as presented in Figure 2b, and gradually formed a dark blue film of tungsten oxide on the substrate, as shown in Figure 2c. Tungsten trioxide nanowires were grown from the protrusions of the film as nucleation points in Figure 2d.

**Figure 3:**
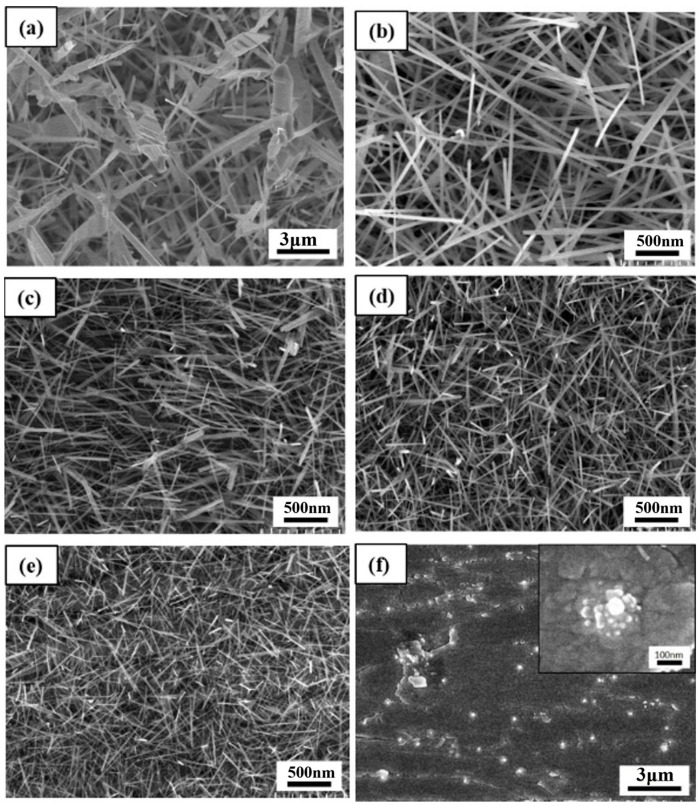
SEM images of NWs grown at (**a**) 850 °C (**b**) 750 °C (**c**) 700 °C (**d**) 650 °C (**e**) 600 °C (**f**) 550 °C at 0.1 torr with 5 sccm Ar for 1.5 h.

In the synthesis, the temperature of the furnace was fixed at 1050 °C. It was found that the amount of precursor residues decreased significantly after the annealing process in the alumina boat below this temperature. Other processing parameters, including substrate temperature and position, pressure, reaction time, and flow rate, were controlled to adjust the diameter and morphology of WO_3_ NWs. Figure 3a–f shows the SEM images of nanowires which were grown at different temperatures (850 °C, 750 °C, 700 °C, 650 °C, 600 °C, 550 °C) for 1.5 h with the pressure of 0.1 torr and carrier gas Ar flow of 5 sccm. Figure 3f reveals the case at 550 °C, where no nanowires but thin films and some gathered points were observed. The diameters of the points were around 20–50 nm, which could be the nucleation sites for nanostructures [23]. In Figure 3e, short nanowires with low densities and diameters of around 40 nm appeared at 600 °C. Figure 3c,d shows the high density and uniformly distributed NWs. The diameter of the NWs in Figure 3c is slightly thinner than that in Figure 3d, ranging from 30–80 nm. In the case of 750 °C, as shown in Figure 3b, the NWs with the highest aspect ratio of about 200 and diameter of 50–100 nm were obtained. With the temperature raised to 850 °C, as presented in Figure 3a, the NWs became much thicker and some of them transformed to rods or dendrites of a diameter ranging over 0.1–1 μm. At a temperature of over 600 °C, the NWs tended to have a lower density but a much longer and slightly thicker morphology. However, when placed at temperature zones higher than 850 °C, the NWs would have enough energy to recrystallize; thus, unfavorable structures such as thick dendrite structures or crystals appeared. The growth rate of the precursor transforming from the vapor state to the solid state can be expressed by the following equation:

(1) $$J=2\pi d_{p}D_{0}C_{m}$$

where *J* is the growth rate, *D*_0_ is the diffusion coefficient between nucleating particles and carrier gas, *d_p_* is the particle size, and *C_m_* is the concentration of the particles. The growth rate is proportional to the particle size and concentration of particles, both of which are temperature-dependent. Also, the particle size can be discussed below:

(2) $$f=4{\left(\frac{\pi kT}{m}\right)}^{1/2}{d_{p}}^{2}N^{2}$$

where *f* is the collision frequency of the particles per unit volume, *N* is the number of particles, and *m* is the mass of the particles. With higher temperatures, the kinetic energy of the particles increases; thus, the collision frequency increases. The temperature is related to the condensation deposition, nucleation, and growth rates of gas molecules. Low temperature decreases the collision frequency, leading to fewer nanowires grown. The two equations above can be used to explain the relationship between temperature and the dimension of the nanowires.

**Figure 4:**
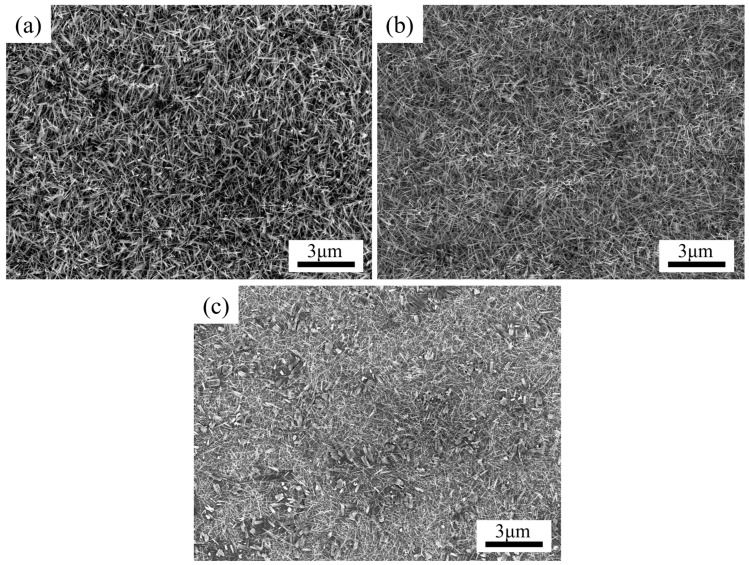
SEM images of NWs grown at carrier gas Ar flow rate of (**a**) 0, (**b**) 5, and (**c**) 50 sccm at 700 °C and 0.1 torr for 1.5 h.

Gas flow rate and deposition position are important parameters in the vapor deposition process [24], which can directly affect the vapor concentration and distribution. Figure 4a–c shows the morphology of NWs grown at 700 °C for 1.5 h while the flow rate of carrier gas Ar was 0, 5 sccm and 50 sccm. Without carrier gas flow, the vapor molecules are diffused to the substrate simply by the concentration gradient [25,26,27]. With some carrier gas flow, the NWs (Figure 4b) were better distributed and more consistent in diameter and length, compared with the NWs of Figure 4a. However, more vapor molecules were taken away from the tube at a higher flow rate; thus, the reaction concentration was low. In this case, fewer precursor molecules were deposited on the substrate, leading to fewer NWs grown, as shown in Figure 4c. Additionally, the diameter of the NWs increased with the higher gas flow rate. This may be associated with the precursor vapor concentration:

(3) $$\Delta Gv=\frac{-kT}{\Omega }ln(C/C_{0})$$

where Δ*G* is the free energy for nucleation, *T* is the temperature, Ω is the atomic volume, *C* is the reaction concentration, and *C*_0_ is the equilibrium reaction concentration. The decrease in the reaction concentration reduces the Δ*G* value in WO_3_, which increases the critical radius of nuclei r* for nucleation, as shown below:

r* = (−2*γ*)/ΔG* (4)

where *γ* is the surface energy, and ΔG* is the critical energy for nucleation. As more Ar gas was introduced, the reaction concentration was lower and the size of the nuclei became larger, which explains why the NWs possess wider diameters.

From the above observation, we found that the growth and morphology of the NWs would be seriously affected by the precursor concentration and growth temperature. Precursor vapor was transported to the W substrate by the carrier gas. At the lower temperature zone, the vapor would first nucleate into small particles. The size of the particles would increase at higher temperatures, and then, the particles would be deposited on the substrate and become a film. The surface of the film was rough with lots of small bulges; thus, as the following particles tried to be deposited, these points would be good nucleation points. With proper control of the growth temperature and concentration, the particles kept nucleating, forming the WO_3_ NWs.

**Figure 5:**
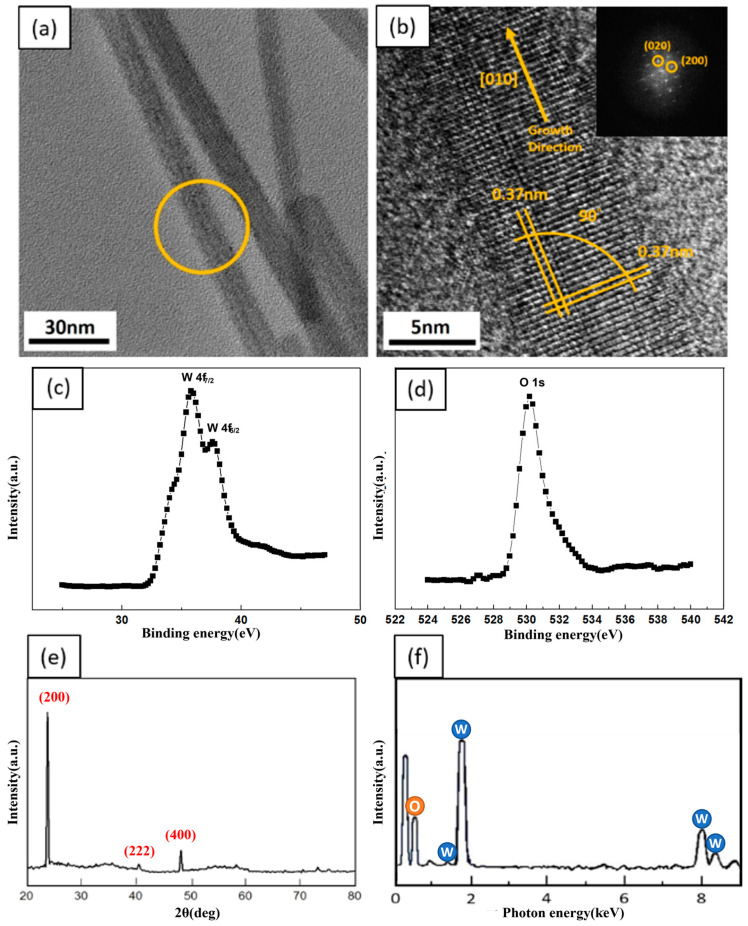
Characterization of WO_3_ NWs grown at 700 °C. (**a**) Low resolution TEM image. (**b**) HR-TEM image and inset is FFT pattern. (**c**) XPS analysis of W4f and (**d**) O1s core levels. (**e**) XRD pattern. (**f**) EDS analysis.

For further analysis of the atomic structure and composition of WO_3_ NWs synthesized at 700 °C, the HR-TEM image, selected-area fast Fourier transform (FFT) pattern and EDX spectrum are given in Figure 5. From Figure 5a, the WO_3_ NWs had no obvious defects on the surface. Figure 5b is the HR-TEM image of yellow circle part in Figure 5a.Through the HR-TEM image and FFT pattern in Figure 5b inset, it is confirmed that the materials were single crystalline WO_3_ NWs of cubic structure with a [010] growth direction. The XRD results given in Figure 5e are consistent with the TEM analysis, showing the diffraction peaks of cubic WO_3_ (JCPDS card No: 46-1096) [28]. Figure 5f reveals the energy-dispersive spectrometry (EDS) results, indicating that the atomic concentrations of W and O are 27% and 73%, which is close to 1:3. X-ray photoelectron spectroscopy (XPS) results for the W4f and O1s core levels are shown in Figure 5c,d [29]. Figure 5d reveals that the peak of O1s is at 530.4 eV, while the two peaks of W4f are at 35.7 eV and 37.5 eV. Additionally, there is a weak peak at the higher binding energy side of the O1s core level that is approximately at 532 eV, which has been discussed in our previous study [30]. The peak results from O^2−^, O^−^ and OH^−^ signals based on oxygen vacancies; this may be attributed to surface contaminants, such as water molecules attached to the specimen or adsorbed oxygen from the air.

### 3.2. Photoluminescence Property

**Figure 6:**
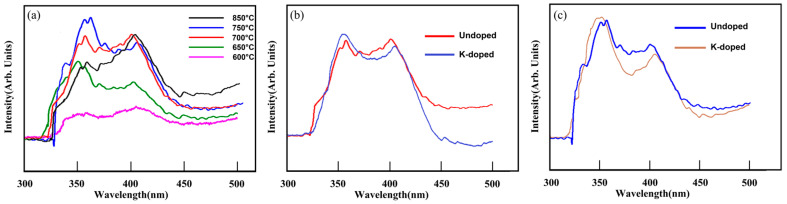
The PL spectrums of WO_3_ NWs synthesized at different temperature zones. (**a**) From 600–850 °C. The undoped and K-doped NWs grown at (**b**) 750 °C and (**c**) 700 °C.

The photoluminescence (PL) property is mainly composed of the near-band-edge emission (3–5 eV) and the defect emission (1.5–2.8 eV). Figure 6 is the PL spectra of WO_3_ NWs synthesized at temperature zones ranging over 600–850 °C. Generally, there are two emission peaks at around 355 and 410 nm. Since the NWs were relatively thin in diameter, the quantum-confinement effects would have great influence on the band gap and induce the near-ultraviolet (NUV) emission peak at 355 nm, which is attributed to the intrinsic band-to-band gap emission. Another broad blue emission peak at 410 nm could result from the oxygen vacancies [31], which is also presented in our previous work [30]. The above two emission peaks are in shorter wavelengths than those in previous studies [32,33].

With the increasing substrate temperature, both the NUV peak and blue emission peak became stronger, which may be related to the density of the NWs [34,35,36]. Notably, the NUV peaks were stronger than the blue emission peak at lower temperature zones, while at higher temperatures, the blue emission peaks became stronger than the NUV peak, such as the 850 °C black line in Figure 6. Since the NWs synthesized at higher temperatures possessed a thicker and more rod-like structure, the quantum-confinement effects had less influence on the band gap, resulting in a lower intensity peak.

Furthermore, we studied the K-doping effect on the PL spectrum of WO_3_ NWs grown at 750 and 700 °C, as given in Figure 6. Both peaks in each sample remained almost the same, and the only difference is that the peaks of the K-doped samples are more separated; this can be attributed to the decreasing surface and crystal defects [37]. Additionally, WO_3_ NWs after the doping process usually possess more defects, such as oxygen vacancies; thereby, the corresponding peak intensity will rise. However, this phenomenon is not observed in Figure 6 possibly because the doping process was conducted at 500 °C for only 20 min; the high temperature contributed to not only the better crystal structure but also fewer vacancies. Thus, after the doping, the intensity of the peak attributed to oxygen vacancies became weaker, as seen in Figure 6.

### 3.3. Field Emission Property

**Figure 7:**
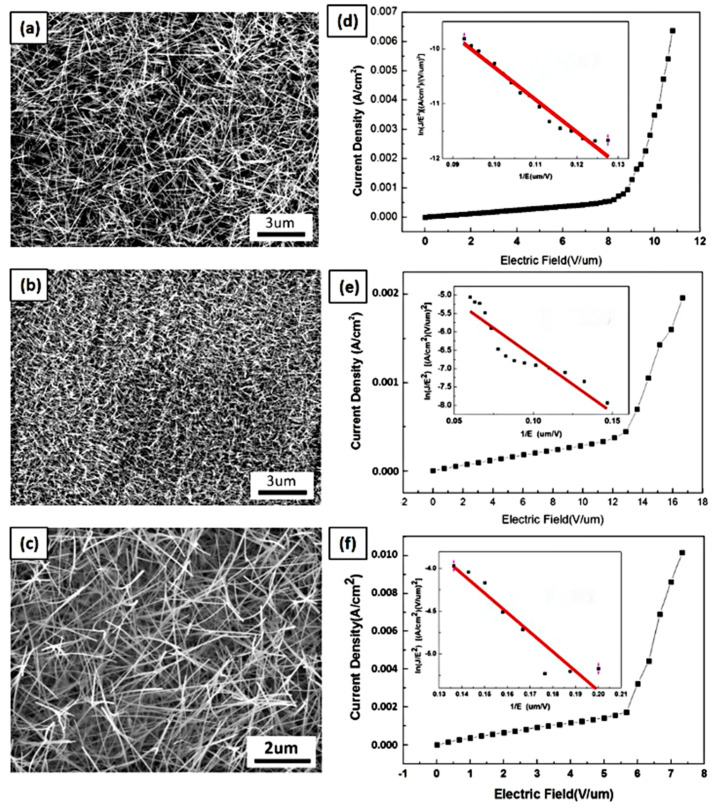
SEM images and field emission measurements of WO_3_ NWs. (**a**,**d**) NWs grown at 750 °C. (**b**,**e**) NWs grown at 650 °C. (**c**,**f**) K-doped NWs grown at 750 °C.

The field emission property is a function of the morphology of the substrate. As for the NWs on the thin film, substrates with a higher NW density, aspect ratio, and proper growth direction will have a better performance. Figure 7a,b shows the measured WO_3_ NWs synthesized at 750 and 650 °C. Both of them were very dense, but the NWs grown at 750 °C had a thicker diameter of around 50 nm, while the NWs grown at 650 °C were 20 nm in diameter. Moreover, the NWs fabricated at 750 °C were so long that they tended to lie down. The NWs grown at 650 °C are expected to show a better field emission property. The J-E curves were the plots of the current density versus the applied electric field in Figure 7d,e, and their turn-on electric fields, defined at the current density of 1 μA·cm^−2^, were 8.5 and 13 V·μm^−1^, respectively. The field emission property can be evaluated by the Fowler-Nordheim equations below:

(5) $$J=\left(\frac{A\beta^{2}E^{2}}{\varphi }\right)exp(-B\varphi^{\frac{3}{2}}/\beta E)$$

conversed to

(6) $$\ln\left(\frac{J}{E^{2}}\right)-\ln\left(\frac{A\beta^{2}}{\varphi }\right)=-B\varphi^{\frac{3}{2}}/\beta E$$

where *J* is the current density in μA·cm^−2^, and *E* is the electric field in V·μm^−1^. A and B are the constants 1.56 × 10^−10^ [AV^−2^(eV)] and 6.83 × 10^3^ [V (eV^−3/2^) (μm^−1^)], respectively, and φ is the work function of 5.7 eV [38,39]. The *β* value can be derived by the Equation (6) corresponding to the slope of the F–N distribution, which was referred to the (*J*/*E*^2^) − (1/*E*) plots. The field enhancement factors of WO_3_ NWs synthesized at 750 °C and 650 °C were 671 and 1268, respectively. Although the turn-on electric field of NWs grown at 650 °C was higher than that of NWs grown at 750 °C, as compared with the previous study [40,41], they had outstanding field emission characteristics caused by their higher aspect ratio and a more vertically-grown morphology. Figure 7c,f shows the SEM image and the F-N curve of the K-doped WO_3_ NWs grown at 750 °C. The threshold electric field decreased to 5.5 (V/um) and the field enhancement factor increased to 1825. The doped potassium atoms and annealing process not only benefited the electrical conductivity of the NWs, but also improved the crystal structure with fewer defects. As a result, K-doped WO_3_ NWs possessed better field emission characteristics than undoped WO_3_ NWs. Table 1 reveals the comparison of the field emission properties of WO_3_ NWs with previous studies. In this work, the K-doped WO_3_ NWs present a lower turn-on electric field and higher field enhancement factor. Furthermore, the WO_3_ NWs here show an excellent field enhancement factor despite their higher turn-on electric field, as compared with previous works. The lengths of the WO_3_ NWs grown here at 650 °C are shorter than those at 750 °C. The shorter the NWs are, the smaller the electric potential is between the bottom of the substrate and the top of the NWs; the stronger electric field would be needed to apply to dissociate electrons from the top of the NWs into the vacuum. Surprisingly, the field enhancement factor increased despite these disadvantages. This is attributed to the significantly higher density, as more NWs provide more electron dissociation points; additionally, the thicker diameter of the nanowires makes it easier for electrons to overcome the energy barrier due to the quantum size effect.

## 4. Conclusions

The high aspect ratio and unique cubic WO_3_ NWs were synthesized by a modified thermal evaporation method, at a relatively high pressure of 0.1 torr with a catalyst-free growth process. The effects of processing parameters, including temperature and gas flow rate, were discussed and controlled to grow the desired morphology. Additionally, the growth mechanism of WO_3_ NWs was proposed. TEM analysis shows WO_3_ NWs with a cubic structure and a growth direction along [010]. The crystal growth planes were identified through XRD analysis. Compared with other cubic structure WO_3_ NWs, the NWs grown at 650 °C had better field emission properties with a high field enhancement factor of 1268; the K-doped NWs grown at 750 °C possessed even better field emission characteristics, with a threshold electric field of 5.5 (V/μm) and β of 1825. Compared with previous studies, the outstanding field emission characteristics are a function of the doping effect, morphology, and lattice structure of the nanowires. The PL spectra were of different intensities for WO_3_ NWs with diverse diameters and morphologies governed by different temperature zones. There were two emission peaks at around 355 and 410 nm, which are related to the intrinsic band-to-band gap emission and oxygen vacancies, respectively. Both NUV and blue emission peaks were at shorter wavelengths than in previous studies; however, there was no shift of the peaks detected after doping. The peaks of K-doped WO_3_ NWs were similar to those of pure WO_3_ NWs; the peaks of the K-doped sample were more separated, which can be attributed to its fewer surface and crystal defects. The high crystallinity and high aspect ratio of the nanowires contributed to their excellent field emission capabilities. The potassium-doped tungsten trioxide nanowires exhibited alterations in their energy band structure, demonstrating their potential as optoelectronic materials.

## Figures and Tables

**Table 1:** Comparison of the field emission properties of WO_3_ nanowires with previous studies.

	Growth Temperature	Growth Method	Turn-on Electric Field	Enhancement Factor	Reference
WO_3_ nanowires	750°C	Thermal evaporation	8.5 V·μm^−1^	671	This work
WO_3_ nanowires	650°C	Thermal evaporation	13 V·μm^−1^	1268	This work
K-doped WO_3_ nanowires	750°C	Thermal evaporation	5.5 V·μm^−1^	1825	This work
WO_3_ nanowires	800°C	Chemical vapor deposition	6.44 V·μm^−1^	691	[40]
WO_3_ nanowires	800°C	Sputter	22 V·μm^−1^	670	[41]

## Data Availability

Not applicable.
